# A multi-layer network approach to MEG connectivity analysis

**DOI:** 10.1016/j.neuroimage.2016.02.045

**Published:** 2016-05-15

**Authors:** Matthew J. Brookes, Prejaas K. Tewarie, Benjamin A.E. Hunt, Sian E. Robson, Lauren E. Gascoyne, Elizabeth B. Liddle, Peter F. Liddle, Peter G. Morris

**Affiliations:** a Sir Peter Mansfield Imaging Centre, School of Physics and Astronomy, University of Nottingham, University Park, Nottingham NG7 2RD, United Kingdom; b Centre for Translational Neuroimaging in Mental Health, Institute of Mental Health, School of Medicine, University of Nottingham, Jubilee Campus, Triumph Road, Nottingham NG7 2TU, United Kingdom

**Keywords:** Multi-layer networks, Magnetoencephalography, MEG, Functional connectivity, Neural oscillations, Schizophrenia, Visual cortex, Motor cortex

## Abstract

Recent years have shown the critical importance of inter-regional neural network connectivity in supporting healthy brain function. Such connectivity is measurable using neuroimaging techniques such as MEG, however the richness of the electrophysiological signal makes gaining a complete picture challenging. Specifically, connectivity can be calculated as statistical interdependencies between neural oscillations within a large range of different frequency bands. Further, connectivity can be computed between frequency bands. This pan-spectral network hierarchy likely helps to mediate simultaneous formation of multiple brain networks, which support ongoing task demand. However, to date it has been largely overlooked, with many electrophysiological functional connectivity studies treating individual frequency bands in isolation. Here, we combine oscillatory envelope based functional connectivity metrics with a multi-layer network framework in order to derive a more complete picture of connectivity *within* and *between* frequencies. We test this methodology using MEG data recorded during a visuomotor task, highlighting simultaneous and transient formation of motor networks in the beta band, visual networks in the gamma band and a beta to gamma interaction. Having tested our method, we use it to demonstrate differences in occipital alpha band connectivity in patients with schizophrenia compared to healthy controls. We further show that these connectivity differences are predictive of the severity of persistent symptoms of the disease, highlighting their clinical relevance. Our findings demonstrate the unique potential of MEG to characterise neural network formation and dissolution. Further, we add weight to the argument that dysconnectivity is a core feature of the neuropathology underlying schizophrenia.

## Highlights

• A multi-layer network framework is applied to MEG envelope connectivity analysis.
• Model allows a complete picture of within and between frequency band connectivity.
• Method is validated using data from a visuomotor task.
• Method shows abnormalities in alpha band connectivity in schizophrenia patients.
• Schizophrenia finding may relate to altered attentional mechanisms in patients.

## Introduction

A core feature of healthy human brain function involves the recruitment of multiple spatially separate and functionally specialised cortical regions, which are required to support ongoing task demand. Such inter-areal connectivity has been shown to be a consistent feature of measured brain activity, even when the brain is apparently at rest (Beckmann et al., 2005, Biswal et al., 1995). Moreover, significant evidence shows that this network formation is altered in pathologies ranging from developmental disorders (e.g. Attention Deficit/Hyperactivity Disorder (Liddle et al., 2011)) to neurodegenerative disease (e.g. Parkinson's disease (Tessitore et al., 2012)) making it a critically important area of study. Measurement and characterisation of networks of functional connectivity is a focus of many neuroimaging studies, and recent years have seen rapid advances in the use of magnetoencephalography (MEG) for this purpose (Engel et al., 2013, Hall et al., 2014, O'Neill et al., 2015a, Scholvinck et al., 2013). MEG (Cohen, 1972) assesses electrical activity in the human brain, based upon measurement of changes in magnetic field above the scalp induced by synchronised neural current flow. MEG offers non-invasive characterisation of brain electrophysiology with excellent temporal resolution. In addition, recent improvements in modelling the spatial topographies of scalp level field patterns allow for spatial resolution on a millimetre scale (Troebinger et al., 2014). This unique combination of high spatial and temporal resolution, coupled with the direct inference on brain electrophysiology, makes MEG a highly attractive option for connectivity measurement, particularly given recent findings that dynamic changes in connectivity occur on a rapid (potentially millisecond) timescale (Baker et al., 2012, Baker et al., 2014, Hutchison et al., 2013, O'Neill et al., 2015b).

Despite its excellent promise, MEG based characterisation of connectivity is complicated by the rich information content of electrophysiological signals. MEG measurements are dominated by neural oscillations (rhythmic changes in electrical potential synchronised across cell assemblies) which occur at multiple temporal scales, ranging from 1 Hz to ~ 200 Hz. These oscillations have been shown to be integrally involved in mediating long range interactions across the cortex (Brookes et al., 2011, de Pasquale et al., 2010, Hipp et al., 2012, Marzetti et al., 2013). However, many studies probe only single frequency bands in isolation without reference to a bigger ‘pan-spectral’ picture. In addition, the richness of the signal facilitates multiple independent measures of functional connectivity (Scholvinck et al., 2013). These include fixed phase relationships between band limited oscillations (Nolte et al., 2004, Stam et al., 2007), as well as synchronisation between the amplitude envelopes of the same band limited oscillations (Brookes et al., 2011, Hipp et al., 2012). Furthermore, evidence shows that in addition to neural interactions within specific frequency bands, connectivity may also be mediated by between frequency band interactions. These might include synchronisation of oscillatory envelopes (Furl et al., 2014) as well as an influence of low frequency phase in one region, on high frequency amplitude in another region (or vice versa) (Canolty et al., 2006, Canolty et al., 2010, Florin and Baillet, 2015). Ongoing mental activity certainly necessitates the simultaneous formation of multiple networks of communication and it seems likely that the brain employs multiple frequency bands, as well as cross frequency interactions and potentially independent modes of connectivity (e.g. phase versus amplitude) in order to achieve this. It therefore follows that a single framework in which to combine pan-spectral and cross frequency interactions to assess the efficiency of the brain as a single multi-dimensional network would be highly desirable.

A potential solution to this problem is a multi-layer network. This concept, which is well studied in physics (see e.g. (De Domenico et al., 2014, Zanin, 2015)), can be understood using the simple example of a transport network. An individual can move between European cities in multiple ways, including by air, rail or road. These three modes of transport can be represented by three seemingly independent networks, with the network nodes being different cities, and the strength of connections between them (i.e. the edges) representing the number of aircraft, trains, or cars that travel between them each day. In order to determine the efficiency of the system, it may be tempting to analyse each network (air, rail, road) in isolation. However, to understand the overall picture, one must also realise that each network depends critically on the other two. For example, a broken rail link between Nottingham and London would increase road traffic between the two cities, and might decrease passengers on flights from London airports. For this reason, a multi-layer network model is required which characterises the three separate networks (air, rail and road) as individual layers in the model, and also measures the dependencies between these networks as between layer interactions. This model allows a more complete characterisation of the overall transport system, taking into account all modes of transport and their interdependencies. This multi-layer framework has been applied to many complex systems, including the human brain. Here we aim to apply it to MEG derived functional connectivity.

In this paper, we use envelope correlation as a means to quantify connectivity between spatially separate brain regions. This metric has been used extensively in recent years (Engel et al., 2013, Hall et al., 2014, O'Neill et al., 2015a) and has been described as an ‘intrinsic mode’ of functional coupling in the human brain (Engel et al., 2013). We estimate ‘all-to-all’ connectivity between a-priori defined brain regions, which are based on an atlas (Tzourio-Mazoyer et al., 2002). Connectivity is estimated within multiple separate frequency bands and these within frequency band interactions define the separate layers in the model (i.e. a single layer is constructed for the alpha, beta and gamma bands independently — these are analogous to the separate road, rail, and air networks described above). Connectivity is also estimated between frequency bands; for example, we might measure correlation between the alpha envelope of brain region 1, and the gamma envelope of brain region 2. This forms the between layer interactions (analogous to interactions between transport modalities (e.g. air to road) in the above example). In this way we aim to form a more complete picture of the brain as a multi-layer dynamic system. In what follows we will test our multi-layer approach on MEG data recorded during a simple visuo-motor task. Further, we will use the same framework to identify perturbed network formation in patients with Schizophrenia.

## Methods

### Data collection

All data used in this study were acquired as part of the University of Nottingham's Multi-modal Imaging Study in Psychosis (MISP; see also Acknowledgements) and have been described in a previous paper (Robson et al., in press). The study received ethical approval from the National Research Ethics Service and all participants gave written informed consent prior to taking part. 23 healthy control subjects (17 male) with no history of neurological illness were recruited to the study. An equal number of patients with schizophrenia were also recruited with the two groups matched for age (Patients: 27 ± 7; Controls 27 ± 7), sex and socio-economic background. In order to derive a score for overall severity of psychotic illness in the patients, the three characteristic syndromes of schizophrenia (reality distortion, psychomotor poverty and disorganization) were quantified (using the signs and symptoms of psychotic illness (Liddle et al., 2002), speed of cognitive processing assessed using a variant of the Digit Symbol Substitution Test and scores from the Social and Occupational Function Scale, respectively). These measurements were combined in a principal component analysis and the first principal component was extracted to give a single score representing the severity of the persistent symptoms of schizophrenia for each patient. We have demonstrated previously that this first component is a suitable measure of severity of residual illness that correlates with several measures of brain function (Palaniyappan et al., 2013, Robson et al., 2015).

All subjects completed a visuomotor task. The paradigm comprised visual stimulation with a centrally-presented maximum contrast vertical square wave grating (3 cycles per degree). The grating subtended a visual angle of 8° and was displayed along with a red fixation cross on a grey background. In a single trial, the grating was presented for 2 s followed by a 7 s baseline period where only the fixation cross was shown. During presentation, participants were instructed to repeatedly press a button with the index finger of their right hand. Participants could press the button as many times as they wanted during the stimulus. A total of 45 trials was used, giving a total experimental time of 7 min. Visual stimuli were back-projected via a mirror system onto a back projection screen inside a magnetically shielded room at a viewing distance of approximately 46 cm. Button presses were recorded using a response pad.

MEG data were acquired throughout the task using a 275 channel CTF MEG system (MISL, Coquitlam, Canada) operating in the third order synthetic gradiometer configuration (Vrba and Robinson, 2001). Data were acquired at a sampling frequency of 600 Hz, and all subjects were oriented supine. Three electromagnetic head position indicator coils were placed on the head as fiducial markers (at the nasion, left preauricular and right preauricular points). The locations of these fiducials were tracked continuously during the recording by sequentially energising each coil and performing a magnetic dipole fit. This allowed both continuous assessment of head movement throughout the measurement, and accurate knowledge of the location of the head relative to the MEG sensors. Prior to the MEG recording, a 3-dimensional digitisation of the subjects head shape, relative to the fiducial markers, was acquired using a 3D digitiser (Polhemus Inc., Vermont). In addition, as part of the MISP programme, all participants underwent an anatomical MRI scan using a Philips Achieva 7 T system. (MPRAGE sequence, volume transmit and 32 channel receive head coil; 1 mm isotropic resolution TE/TR = 3/7 ms; FA = 8°). Coregistration of the MEG sensor geometry to the anatomical MR image (hence brain anatomy) was subsequently achieved by fitting the digitised head surface to the equivalent head surface extracted from the anatomical MR image. This coregistration was employed in all subsequent forward and inverse problem calculations.

### Data analysis

**Fig. 1:**
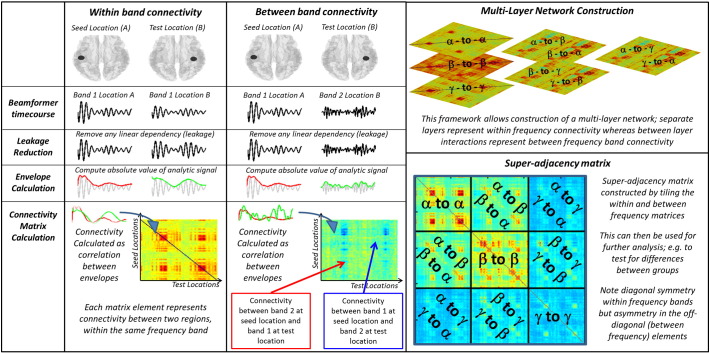
Schematic diagram of the connectivity data analysis pipeline including construction of a multi-layer network. Note that, in our actual analysis, the gamma band was split into two, separating low gamma (30 Hz–50 Hz) and high gamma (50–100 Hz). However in order to simplify the Figure, this is not shown.

MEG data were initially inspected visually. Any trials deemed to contain an excessive amount of interference, for example generated by eye movement or muscle activity, were removed from that individual's data. In addition, any trials in which the head was found to be more than 7 mm (Euclidean distance) from the starting position were excluded. Following this pre-processing, data were analysed using beamforming for source localisation, and a multi-layer network framework (see also Fig. 1).

#### AAL atlas and source localisation

In order to calculate a whole cortex representation of functional connectivity, the cortex was first parcellated into 78 individual regions according to the automated anatomical labelling (AAL) atlas (Tzourio-Mazoyer et al., 2002). Note that these same cortical regions have been used successfully in previous MEG connectivity studies (see e.g. (Tewarie et al., 2014)). A beamformer spatial filtering approach (Robinson and Vrba, 1998) was then employed to generate a single signal representative of electrophysiological activity within each of these 78 regions. To achieve this, for each region, the centre of mass was derived. Voxels were also defined on a regular 4 mm grid covering the entire region, and the beamformer estimated timecourse of electrical activity derived for each voxel. To generate a single regional timecourse, ${\hat{Q}}_{R}\left(t\right)$, individual voxel signals were weighted according to their distance from the centre of mass such that,

(1) Q^Rt=∑iexp−ri2400Q^it

where *i* represents a count over all voxels within the AAL region, ${\hat{Q}}_{i}\left(t\right)$ represents the beamformer projected timecourse for voxel *i*, and *r*_*i*_ denotes the distance (measured in millimetres) from voxel *i* to the centre of mass of the region. Note that the Gaussian weighting function ensures that the regional timecourse ${\hat{Q}}_{R}\left(t\right)$ is biased towards the centre of the region. The full width at half maximum of the weighting was ~ 17 mm; this was chosen to reflect the approximate spatial scale of the AAL regions.

To calculate the individual ${\hat{Q}}_{i}\left(t\right)$, a scalar variant of beamforming was employed (Robinson and Vrba, 1998). Covariance was computed within a 1 Hz–150 Hz frequency window and a time window spanning the whole experiment in order to minimise covariance matrix error (Brookes et al., 2008). Regularisation was applied to the data covariance matrix using the Tikhonov method with a regularisation parameter equal to 5% of the maximum eigenvalue of the unregularised covariance matrix. The forward model was based upon a dipole approximation (Sarvas, 1987) and a multiple local sphere head model (Huang et al., 1999). Dipole orientation was determined using a non-linear search for optimum signal to noise ratio (SNR). Beamformer timecourses were sign flipped where necessary in order to account for the arbitrary polarity introduced by the beamformer source orientation estimation.

#### Regional changes in source amplitude

Application of the beamforming method to each AAL region yielded 78 regional timecourses and we initially aimed to assess which of those timecourses (hence regions) exhibited a significant task induced response. Regional timecourses were frequency filtered into four separate frequency bands; alpha (α) (8 Hz–13 Hz), beta (β) (13 Hz–30 Hz), low gamma (γ_L_) (30 Hz–50 Hz) and high gamma (γ_H_) (50 Hz–100 Hz). The resulting timecourses were then Hilbert transformed in order to generate the analytic signal. The absolute value of the analytic signal was then computed to yield the amplitude envelope (henceforth termed the Hilbert envelope) of each timecourse. Hilbert envelopes were averaged across trials. In order to determine the AAL regions that exhibited a significant task related power change, the fractional change in oscillatory amplitude (for all frequency bands and regions) was measured between a ‘stimulus’ window [0 s < t < 2 s] and a ‘rebound’ window [2 s < t < 4 s]. (These windows were chosen to give maximum contrast in the motor system, since the stimulus window will centre on movement related beta power decrease whereas the rebound window will centre on the post movement beta rebound.) The statistical significance of the fractional change between windows was determined using a two-sided signed rank test of the null hypothesis that the change in Hilbert envelope (measured independently in 23 subjects) originated from a distribution whose median is zero. The threshold for significance (p < 0.05) was Bonferroni corrected to account for multiple comparisons across all 78 regions. In four AAL regions of interest (left sensorimotor cortex, right sensorimotor cortex, left primary visual cortex and right primary visual cortex) a time frequency spectrogram was generated. Again this employed the Hilbert transform, however in order to increase spectral resolution, Hilbert envelopes were generated in 33 overlapping frequency bands in the 1 Hz to 150 Hz range. Hilbert envelopes were averaged across all trials and then concatenated in the frequency dimension to form a time-frequency spectrogram (TFS) for the average trial. These TFSs were then averaged across subjects.

#### The multi-layer model: Leakage correction, muscle artifact reduction, and connectivity estimation

The overall aim of our connectivity analysis was twofold. First, to examine significant changes in functional connectivity induced by the visuomotor task in healthy individuals. Second, to probe differences in functional connectivity between schizophrenia patients and controls. To achieve these aims, all connectivity analyses were applied within predefined time windows, on a trial by trial basis, using unaveraged beamformer projected data (i.e. following beamforming we measured amplitude envelope correlation within specific time windows for each trial, and then averaged these connectivity estimates across trials. Time windows were defined as follows:

• To examine task induced change in healthy controls, we measured connectivity within an active [0 s < t < 4 s] window and a control [4.5 s < t < 8.5 s] window; contrasting the two windows in order to derive significant connectivity change. Note that the active window was selected such that it encompassed both the stimulus, and any post stimulus response (e.g. the rebound in the motor regions). In addition, note that the longer the time window used, the more reliable the connectivity estimate becomes. For this reason the two windows were made as long as possible and equal in length to allow for robust and unambiguous contrast.
• To compare controls to patients with schizophrenia, we measured connectivity across the whole trial using a [0 s < t < 8.5 s] window. This was done separately in the two groups (patients and controls) and results compared.

In all cases, functional connectivity was computed between every pair of AAL regions (henceforth known as the seed and the test region). Regional timecourses were again frequency filtered into four separate frequency bands; alpha (α) (8 Hz–13 Hz), beta (β) (13 Hz–30 Hz), low gamma (γ_L_) (30 Hz–50 Hz) and high gamma (γ_H_) (50 Hz–100 Hz). These bands were chosen based upon previous literature; specifically, previous work has shown robust effects in visual cortex in the alpha and gamma bands (Brookes et al., 2005, Zumer et al., 2010) as well as robust effects in motor cortex in the beta band (Stancak and Pfurtscheller, 1995, Stevenson et al., 2011). A schematic diagram of the multi-layer framework is shown in Fig. 1; note however that for simplicity we only depict 3 frequency bands.

Estimation of electrophysiological functional connectivity is non-trivial and warrants some discussion. The most significant confound in MEG connectivity analysis is that of signal leakage between beamformer projected timecourses. This is generated as a result of the ill-posed inverse problem and means that projected timecourses can be artifactually correlated. This problem, and associated solutions, have been well documented in the literature (Brookes et al., 2012, Colclough et al., 2015, Hipp et al., 2012, Maldjian et al., 2014, O'Neill et al., 2015a). Here we employed a pairwise leakage reduction scheme (Brookes et al., 2012, Hipp et al., 2012) which exploits the fact that leakage manifests as zero-time lag correlation between beamformer projected timecourses from separate regions. Such zero-time lag linear dependency was removed using linear regression to ensure that, prior to connectivity estimation, the underlying band limited windowed signals were orthogonal. It is important to note that, in studies of this type where separate time windows are to be compared, orthogonalisation must be carried out on each window separately, rather than on the whole timecourse, since task induced changes in signal variance can also introduce significant changes in the magnitude of leakage (see the analytical analysis in the supplementary information from (O'Neill et al., 2015b)).

Following leakage reduction, the Hilbert envelope was computed for the orthogonalised seed and test timecourses. In addition to leakage, artifacts due to muscle activity were also a concern, particularly for high gamma band connectivity estimation. It is well known that increased muscle activity in, for example, the jaw or neck, generates increased oscillatory signals in the high gamma band. Such artifacts are typically bilateral and can cause spurious inflation of interhemispheric gamma envelope correlation. For this reason, the regional beamformed timecourses were also filtered into the 120 Hz–150 Hz band. This band was deemed to be higher than any neural activity of interest but would accurately capture any artifacts resulting from the magnetomyogram. Prior to calculation of connectivity, the Hilbert envelope of these magnetomyogram data was computed and regressed from both the seed and test timecourses (independently for each trial) in order to reduce the influence of muscle artifact on functional connectivity measurement (see also (O'Neill et al., 2015b) who use a similar method).

Following leakage and magnetomyogram reduction, connectivity was calculated between windowed timecourses as the Pearson correlation coefficient between windowed oscillatory envelopes in the seed and test regions. As noted above, correlation coefficients were computed within each time window, and each trial separately, and the mean correlation coefficient over all trials computed. This same procedure was applied:

1. Within each frequency band (i.e. within the alpha, beta, low gamma and high gamma bands) and between each region pair. This generated four 78 × 78 adjacency matrices (AMs) showing inter-regional connectivity for each of the four bands separately. These formed the 4 separate layers of the multi-layer model (see the “within band connectivity” column of Fig. 1).
2. Between each pair of frequency bands (i.e. alpha-to-beta, alpha-to-low-gamma, alpha-to-high-gamma, beta-to-low-gamma, beta-to-high-gamma and low-gamma-to-high-gamma) and between each region pair. This generated a further six 78 × 78 AMs showing inter-regional connectivity for each of the six frequency band pairs. These formed the between layer interactions of the multi-layer model (see the “between band connectivity” column of Fig. 1).

These processes yielded a total of 10 adjacency matrices (which we also term tiles). These were combined to generate a single ‘super-adjacency matrix’ (SM), an example of which is shown in Fig. 1. The SM contains a complete description of both within frequency band and between frequency band connectivity, measured across the entire brain. A single SM was generated for each time window (active, control and whole trial), meaning that three separate SMs were available for each subject. Contrasting these separate SMs allows testing for differences in network connectivity between task and rest, or patient and control. Note that the individual tiles making up the SM have different symmetries: The within frequency band matrices (diagonal tiles) have diagonal symmetry, since correlation, for example, between visual alpha and motor alpha, is identical to correlation between motor alpha and visual alpha (i.e. the two calculations commute; order doesn't matter). However, this diagonal symmetry is not reflected in the off diagonal tiles (between frequency AMs). This is because a high correlation between, for example, visual alpha and motor gamma does not necessarily imply a high correlation between visual gamma and motor alpha.

#### The multi-layer model: statistical testing for task induced connectivity changes

To test for an effect of the visuomotor task on connectivity, we contrasted SMs measured in the [0 s < t < 4 s] active and the [4.5 s < t < 8.5 s] control time windows. This was done via subtraction, generating a single matrix for each subject showing the difference in connectivity between time windows. These difference-SMs (dSMs) were then averaged across subjects. In order to assess statistical significance, a permutation test was employed (Nichols and Holmes, 2001). It was reasoned that if the task had no effect, then the labelling of the two time windows (active or control) would have no meaning. For each element in the SM, we therefore constructed a null distribution. This was calculated via the generation of multiple ‘sham’ dSMs where the window labels were switched randomly. 20,000 sham matrices were constructed and a null distribution of connectivity differences derived. For each dSM element, the ‘real’ difference between windows was compared to the null distribution and a p-value generated. In order to correct for type I errors due to multiple comparisons across matrix elements, we applied a false discovery rate (FDR) correction based on the Benjamini–Hochberg procedure. This procedure resulted in a thresholded dSM showing which connectivity values in the dSM were modulated significantly by the task.

#### The multi-layer model: testing for differences in patients with schizophrenia

In the case of testing for effects of schizophrenia on connectivity, we employed SMs generated using a single time window spanning the whole trial [0 s < t < 8.5 s]. In order to probe the relevance of our connectivity measurements to schizophrenia, two tests were used.

• First it was reasoned that if connectivity was abnormal in schizophrenia, then a difference between mean connectivity values across the patient and control groups would be observed. This is henceforth termed the *effect of diagnosis* and was measured by subtraction of patient and control SMs.
• Second, it was reasoned that if such a difference was meaningful clinically, then connectivity values measured within individual patients would correlate significantly with their severity of symptoms (measured behaviourally — see above). This is henceforth termed *effect of severity* and was measured, on an element by element basis, by Pearson correlation (across all 23 patients) between severity and estimated connectivity in each element of the SM.

These two tests yield two new matrices, both equal in size to the SM, which represent the *effect of diagnosis* and the *effect of severity*.

Under a null hypothesis that there is no systematic effect of either diagnosis or severity on functional connectivity measurements, then it would be predicated that there would be no significant relationship, across elements, between matrices representing diagnosis and severity. However, if the MEG connectivity measures are truly descriptive of schizophrenia, then those matrix elements most affected by the patient–control difference might be expected to be the same elements that are most correlated with severity. Hence a relationship between the diagnosis and severity matrices would be observed. With this in mind, we measured correlation across matrix elements, on a ‘tile-by-tile’ basis (henceforth termed *tile correlation*). (In other words, we first correlate elements from the tile representing alpha-to alpha connectivity, then do the same thing with the tile representing alpha to beta connectivity, and so on, for all 10 independent tiles.) To test this statistically we used a permutation test. First, patient/control labels were switched randomly and a new average difference between sham groups computed. (This was based on the assumption that if diagnosis has no effect on connectivity then patient and control labels would be meaningless). Second, the individual patient disease severity scores were randomised across subjects and the correlation with connectivity score recomputed. (Based on the assumption that if connectivity had no effect on severity then the re-ordering of the severity scores would have no effect). This yielded two ‘sham’ matrices which could be compared, and again we measured tile correlation. 10,000 iterations of this test were used to generate a null distribution and comparison with the ‘real’ tile correlation value yielded a probability that the result occurred by chance. We used a two tailed test: meaning that we allow the possibility that those patients with the worst symptoms could look more like controls than patients with lesser symptoms — though apparently counter-intuitive such an effect is conceivable and could result from compensation mechanisms. Finally, since testing each tile individually led to 10 separate tests, Bonferroni correction was performed. Statistical significance was therefore defined at a threshold of p < 0.05, which is corrected to p < 0.0025 to account for the two tailed test and 10 separate comparisons. Anything at p < 0.025 (i.e. uncorrected for multiple comparisons) was considered a ‘trend’. It should be noted here that, in principle, a standard parametric test (distinct from our permutation approach) could also be employed; however this would require direct estimation of the degrees of freedom in the correlation. The spatial smoothness inherent in the tiles of the SM means that the number of degrees of freedom in the correlation is vastly less than the number of matrix elements (78^2^). Estimating the reduction in degrees of freedom, whilst possible, is non-trivial. For this reason we employ the permutation approach, where spatially smoothness in the measured tiles is also mirrored in the sham tiles.

The tile correlation test was used to identify tiles in the SM in which connectivity values were related significantly to schizophrenia. (i.e. tiles in which the effect of diagnosis correlated with the effect of severity). Following this, tiles deemed significant were used in order to visualise which individual brain connections were driving the observed significant correlation. To do this, for each matrix element within a significant tile, we first measured the effect of diagnosis (tested using a permutation test); second we measured the effect of severity (again tested via permutation). These tests were treated independently and those matrix elements significant (p < 0.01) in both tests were used in visualisation.

## Results

### Task induced changes in brain activity and connectivity

**Fig. 2:**
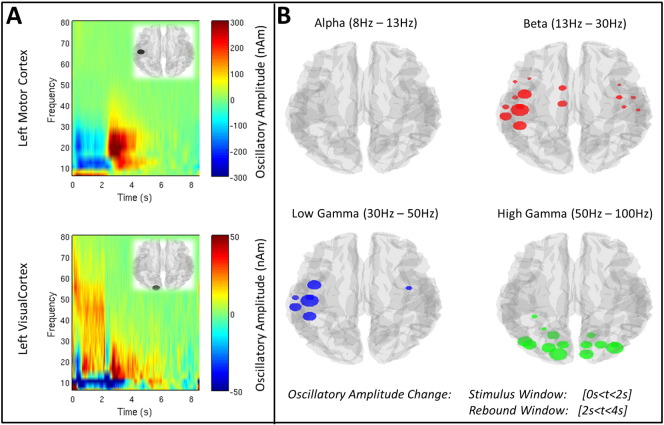
Task induced changes in oscillatory amplitude. A) TFSs generated in the left primary sensorimotor region, and left primary visual area, in healthy control subjects. B) AAL regions exhibiting a significant (p_c_ < 0.05) change in oscillatory amplitude between stimulus and rebound windows. The four images show the four separate frequency bands studied (alpha, beta, low gamma and high gamma).

Fig. 2 shows the change in oscillatory amplitude induced by the visuomotor task. Fig. 2A shows time frequency spectrograms (TFSs) extracted from the left primary sensorimotor cortex (upper panel), and left primary visual cortex (lower panel). Note that, as expected, in sensorimotor cortex a reduction in beta amplitude is observed during stimulation with an increase above baseline immediately following movement cessation. In visual cortex, an increase in gamma amplitude is observed during stimulation alongside a concomitant decrease in alpha amplitude. These results are further shown in Fig. 2B, where the coloured circles show the locations of AAL region centroids with a significant (p_c_ < 0.05) change in neural oscillatory amplitude between stimulus and rebound windows. The sizes of the circles reflect the magnitude of the change. Note that significant changes are observed in motor cortex for beta and low gamma bands, and in visual cortex in the high gamma band.

**Fig. 3:**
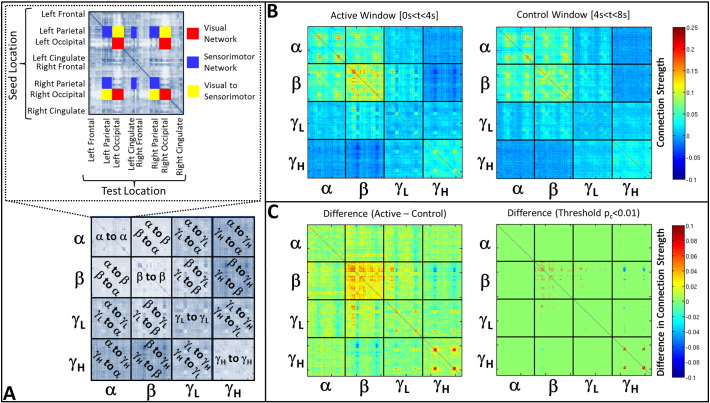
Task induced change in functional connectivity. A) Schematic showing structure of each individual tile (upper panel) and how these are combined to form the super-adjacency matrix (lower panel). B) Super-adjacency matrices computed in the active (left) and control (right) time windows. Matrices show within frequency band (diagonal tiles) and between frequency band (off diagonal tiles) interactions. C) Task induced change (Active–Control) in connectivity. The left hand panel shows change averaged across all subjects. The right hand panel shows the same matrix thresholded to include only statistically significant (p_c_ < 0.01 – FDR corrected) changes in connectivity. Note the main differences occur in the beta and high gamma bands, with significant between frequency interactions in the beta to low gamma, and beta to high gamma bands.

**Fig. 4:**
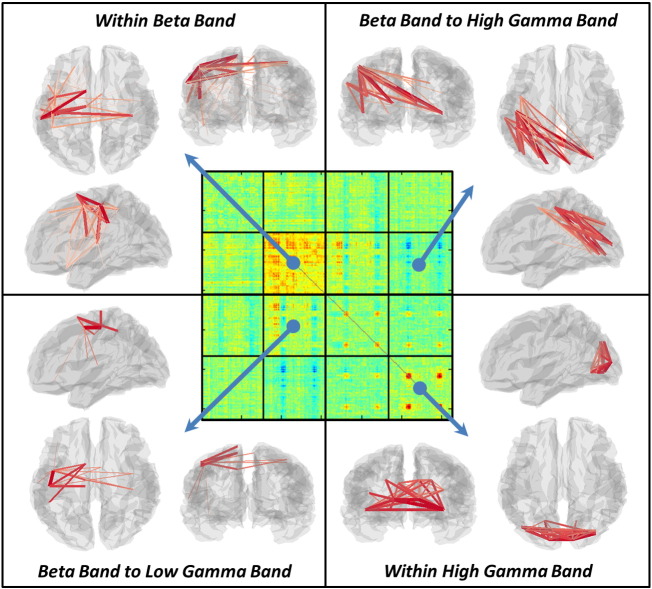
Visualisation of task induced change in functional connectivity. The central matrix depicts the dSM, whilst the outer images show significant task induced changes within individual tiles. Significant results are observed in the beta and high gamma bands, as well as between frequency band effects in the beta to low gamma, and beta to high gamma ranges. In all images, the line thickness represents the magnitude of task induced connectivity change.

Fig. 3, Fig. 4 show task induced change in functional connectivity. Firstly, Fig. 3A presents a schematic diagram showing the structure of each individual adjacency matrix tile (upper panel) and how these tiles are used to form the Super adjacency matrix (lower panel). In the upper panel, regions of the adjacency matrix corresponding to the visual, motor and visual-to-motor networks are highlighted in red, blue and yellow respectively.

Fig. 3B shows SMs, averaged across all subjects, in the active (left) and control (right) time windows. Note first that a high degree of structure is observable in both matrices, particularly in the alpha and beta bands. Note also that, particularly in high frequency bands, increased structure is observable in the active compared to the control window. These results are further confirmed in Fig. 3C which shows the average difference between active and control windows (left) and the thresholded (p_c_ < 0.01 — FDR corrected) difference (right). Comparison of the individual tiles of Fig. 3B and 3C with the upper panel of Fig. 3A show clearly that visual networks are observed in the alpha and gamma bands, alongside a sensorimotor network in the beta band. Note also an anti-correlation between motor cortex beta oscillations and visual cortex high gamma oscillations. This manifests as significant clusters in the beta to high gamma band tile. Note the asymmetry meaning that a reciprocal ‘motor gamma to visual beta’ network is not observed.

Fig. 4 shows visualisation of the transient brain networks formed during the active window of the visuo-motor task. The central panel shows the dSM, and in the outer images, red lines denote the connections between brain region pairs that exhibit a significant task induced change in functional connectivity. The thickness and colour of the line denotes the strength of connection. Within frequency band changes are observed in the beta and gamma ranges. The beta band shows a transient task induced increase in connectivity within a motor network. Specifically, connectivity is increased between the left and right primary motor regions as well as between left primary motor cortex, pre-motor cortex, supplementary motor area (SMA) and the left secondary somatosensory area (S2). This finding is in good agreement with previous results in motor tasks (for example (O'Neill et al., 2015b)). The high gamma band also demonstrates increased connectivity in a visual network which includes primary visual regions and associated (lateral) visual areas. Again this is in good agreement with the well-known effect of increased gamma oscillations with presentation of visual gratings (Adjamian et al., 2004, Hall et al., 2005, Zumer et al., 2010). Significant between frequency band interactions are also observed. Beta to low gamma band connectivity is increased during the task within a network of brain areas which includes bilateral pre-motor cortex and left primary motor cortex. Note the spatial difference between this beta to low gamma band interaction and the beta network, the former being centred on premotor regions whilst the latter is centred on primary motor cortices, making it tempting to speculate that these networks perform different functional roles. Finally, a beta to high gamma band *reduction* in connectivity is observed between the visual cortex and the left sensorimotor region. These effects will be addressed further in our discussion.

### Difference between patients with schizophrenia and controls

**Fig. 5:**
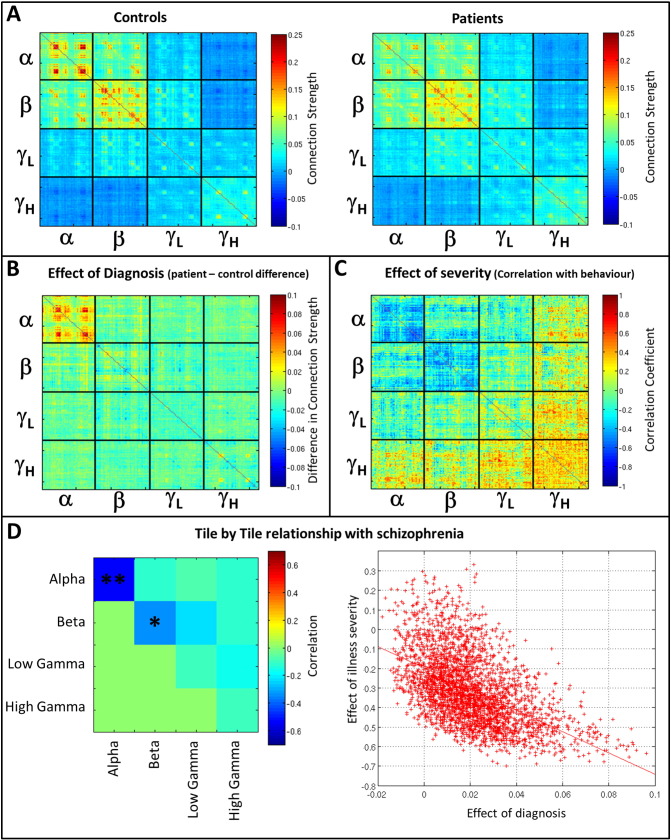
Differences in functional connectivity between controls and schizophrenia patients. A) Super-adjacency matrices computed in controls (left) and patients (right). B) Effect of diagnosis (i.e. difference in connectivity between groups (Controls–Patients)). C) Effect of severity (correlation across individuals between connectivity and the severity of persistent symptoms of schizophrenia, measured by questionnaire). D) Tile correlation showing the relationship between the effects of diagnosis and effects of severity. Relationships are measured as Pearson correlation coefficients across all matrix elements within each tile of the super-adjacency matrix. ** indicates a significant correlation (p_c_ < 0.05 corrected for multiple comparisons across tiles). * indicates a trend (p < 0.05 uncorrected). The right hand panel shows the single example of correlation across matrix elements in the alpha band (p = 0.0005).

Fig. 5 shows the effects of schizophrenia on multi-layer network connectivity. Fig. 5A shows the mean SMs computed in controls (left) and patients (right). Recall that these matrices are computed within a single window spanning the entire length of the task trial, with connectivity estimated for each trial separately and averaged across trials, and subsequently subjects. Fig. 5B shows the difference between groups (Controls–Patients) which we term the effect of diagnosis. Note that clear structure in the difference matrix is observable, particularly within the tile representing alpha-to-alpha connectivity. Fig. 5C shows the cross subject correlation (patients only) between functional connectivity and the severity of persistent symptoms of schizophrenia, which we term the effect of severity. Again a clear structure is observable, particularly in the alpha-to-alpha tile. Under a null hypothesis where connectivity metrics are unaffected by illness, then the effect of diagnosis (i.e. the matrix in Fig. 5B) and the effect of severity (i.e. the matrix in Fig. 5C) would be completely unrelated and show no similarity. However, visually it is easy to see a clear relationship within some (but not all) tiles within these matrices. Fig. 5D formalises this relationship: each element in the matrix represents tile correlation between effect of diagnosis and effect of severity. Relationships are measured as Pearson correlation coefficients across all matrix elements within each tile (see Fig. 5D for an example of alpha-to-alpha connectivity). Notice that, as would be expected from Fig. 5B and 5C, alpha-to-alpha connectivity shows a significant relationship between effects of diagnosis and severity, implying that these connectivity estimates are affected by schizophrenia. Interestingly, no other tiles show a significant relationship following multiple comparison correction.

**Fig. 6:**
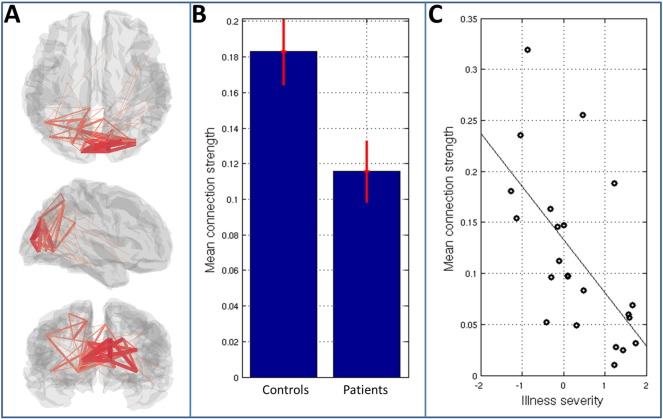
Visualisation of the differences in alpha band functional connectivity between patients and controls. A) Shows the brain regions between which connectivity differs most between groups. The line width represents the strength of the difference. B) Mean connection strength, averaged across the network identified in (A), for patients and controls. Error bars represent standard error across subjects. C) Mean connection strength (again averaged over all connections in (A)) computed in 23 patients and plotted against a measure of illness severity.

Having shown a significant effect of schizophrenia within alpha-to-alpha connectivity, we further investigate these effects in Fig. 6. Fig. 6A highlights the brain regions between which connectivity differs (in terms of both diagnosis and severity) between groups. Again the lines denote connectivity between AAL regions and their width indicates the magnitude of the difference between patients and controls. Note that a clear network structure is observed with the occipital lobe being most strongly implicated. Fig. 6B shows mean connection strength, averaged across the observed occipital network, in both patient and control groups. The bar chart shows mean group connectivity and error bars represent standard error across subjects. Fig. 6C shows mean connection strength (again averaged over all connections in the occipital network) computed separately in 23 patients and plotted against illness severity. Note how, in patients with less severe symptoms, alpha band connectivity tends to a value close to that of controls, whereas in those patients with more severe symptoms, the mean alpha band connectivity is markedly reduced. This important point implies direct clinical relevance of the results shown, which will be further addressed in the discussion below.

**Fig. 7:**
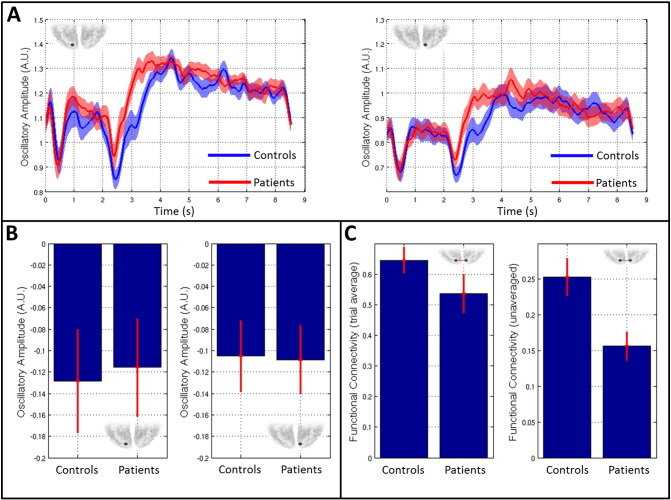
Alpha band amplitude and connectivity changes in visual cortex. A) Timecourses of alpha band oscillatory envelope in patients (red) and controls (blue). The left hand plot shows left visual cortex whereas the right hand plot shows right visual cortex. B) The left and right bar charts show mean task induced change in alpha band oscillatory amplitude in left and right visual cortices respectively. C) The left hand plot shows alpha “connectivity” between left and right visual cortices, calculated using trial averaged data (i.e. correlation between the trial averaged alpha envelopes). The right hand bar chart shows alpha connectivity between left and right visual regions based on unaveraged data. Note a significant difference in connectivity when calculated using unaveraged data. This is measured in the absence of measurable differences in task induced amplitude change or a significant change in trial averaged correlation.

Finally, Fig. 7 shows results of a post-hoc analysis of primary visual cortex activity and connectivity in the alpha band. Fig. 7A shows timecourses of alpha band Hilbert envelope, averaged over trials and subjects. The blue line shows the mean alpha envelope for controls whereas red shows the equivalent envelope in patients. The left hand plot shows the case for left visual cortex and the right hand plot shows right visual cortex. Note that there is relatively little difference in trial averaged alpha envelopes between patients and controls; both groups exhibit marked alpha desynchronisation during stimulation with the largest changes from baseline occurring shortly after stimulus onset and offset. The similarity of the trial averaged alpha band envelopes is further confirmed in Fig. 7B. Here, the left and right bar charts show mean change in alpha amplitude between a stimulus window [0 s < t < 2 s] and a control window [6.5 s < t < 8.5], in left and right visual cortices respectively. Note that amplitude is reduced during stimulation; however there is no measurable difference between patients and controls. Fig. 7C shows alpha connectivity measured between left and right visual regions. In the left hand plot, distinct from the rest of this study, “connectivity” is measured between trial averaged Hilbert envelopes; i.e. the bar chart reflects correlation between the trial averaged alpha band Hilbert envelopes measured in left and right visual cortex. [This is measured independently in each subject and the result averaged across subjects; error bar shows standard error.] In the right hand plot, connectivity is measured using the standard method in unaveraged data (i.e. envelope correlation is measured within each trial and these correlation values are subsequently averaged across trials — as described in our Methods section). Note that a significant difference in connectivity is observed between groups in the unaveraged case, but not in the averaged case. Averaging across trials prior to connectivity estimation causes a marked reduction in any signal fluctuations that are not time locked to the stimulus — meaning that trial averaged “connectivity” is a reflection of the degree to which task induced change is coordinated between regions. It thus follows that the reduction in alpha connectivity observed in Fig. 5, Fig. 6 is not due to atypical coordination of the task induced response between regions; rather, the primary effect is due to the superposition of atypical task independent activity that that fails to synchronise between regions. This will be addressed further below.

## Discussion

Recent years have shown the critical importance of inter-regional neural network connectivity in supporting healthy brain function. Such connectivity is measurable using neuroimaging techniques such as MEG, however the richness of the electrophysiological signal makes gaining a complete picture challenging. Specifically, connectivity can be calculated as statistical interdependencies between neural oscillations measured across a large range of frequencies, as well as between frequency bands. This pan-spectral nature of network formation likely helps to mediate the simultaneous formation of multiple brain networks, which support the demands of ongoing mental tasks. However, to date, in studies of electrophysiological connectivity this has been overlooked, with many studies treating individual frequency bands in isolation. Here, we combine envelope correlation based assessment of functional connectivity with a multi-layer network model in order to derive a more complete picture of connectivity within and between frequency bands. Using a visuomotor task, we have shown that our method can highlight simultaneous and transient formation of a motor network in the beta band, and a visual network in the high gamma band. More importantly, we have used this same methodology to demonstrate significant differences in occipital alpha band functional connectivity in patients with schizophrenia relative to controls. This methodology represents an improved means by which to obtain a more complete picture of network connectivity, whilst our findings in schizophrenia demonstrate the critical importance of measuring connectivity in clinical studies.

### Methodology and the visuomotor task

Methodologically, this paper demonstrates the utility of a multi-layer model in characterising within and between frequency interactions. In our visuomotor application, it was our intention to demonstrate this framework using a well characterised task that is known to induce robust changes in neural oscillations in multiple frequency bands. It is well known that finger movement induces a drop in beta band oscillatory amplitude in primary sensorimotor cortex during movement, followed by an increase above baseline shortly following movement cessation. Furthermore, it is also known that beta band envelopes are associated with long range motor network connectivity. Here we added to this picture by showing directly that unilateral finger movement is supported by the transient formation of a broad network of brain regions including left and right primary motor cortices as well as pre-motor cortices, SMA and secondary somatosensory regions; further, this network is mediated in the beta band. In addition, passive viewing of a visual grating has long been known to increase the amplitude of gamma oscillations in primary visual cortex (Adjamian et al., 2004, Hall et al., 2005). Here we have shown that induced gamma envelopes are correlated across visual regions. Whilst this interaction may be expected, it is interesting to note that it is not simply due to signal leakage between hemispheres. Linear interactions (i.e. simple zero-phase lag correlation between signals measured at spatially separate locations) have been removed via our leakage reduction methodology. The significant increase in connectivity observed therefore represents envelope correlation mediated by non-zero phase lagged (i.e. time lagged) events in the underlying neural signals. To the authors' knowledge this is the first direct measurement of this effect, which may warrant further investigation in future studies. Finally, significant task driven changes between frequency bands were also observed. A network involving bilateral pre-motor and left primary motor areas was observed as a beta to low gamma interaction and the spatial differences noted between this and the motor network limited to the beta band makes it tempting to speculate that the cross frequency interaction serves a different functional role, however this requires significant further investigation. An anti-correlation between the motor and visual regions was also measurable as a beta to high gamma interaction. Whilst it may be tempting to interpret this as a network that coordinates activity between these two regions, it should be pointed out that, given the task is well known to increase gamma amplitude and simultaneously decrease beta amplitude in the visual and motor areas respectively, such an interaction would be expected. In fact, the likelihood is that this transient anti-correlation results from two independent stimulus driven variations, rather than a functional network *per se*. This said however, this cross frequency network also potentially warrants further investigation. Overall, despite some ambiguity, the visuomotor task represents a useful testbed for the multi-layer network framework and its ability to extract simultaneous transiently forming networks both within and across frequency bands.

In terms of the method itself, there are four core components that warrant discussion: cortical parcellation; source space projection; the connectivity metric and statistical analysis. First, regarding the AAL parcellation, this was chosen based on its successful use in previous MEG investigations (e.g. (Tewarie et al., 2016, Tewarie et al., 2014)). However, our method could be used with any cortical parcellation. It is noteworthy that the separate AAL regions vary markedly in size, meaning that our use of a single full width at half maximum of the Gaussian function (Eq. (1)) may mean that some regions are better represented than others; this represents a limitation of the present method. Related, the inhomogeneous spatial resolution of MEG may mean that, in some cases multiple AAL regions may generate degenerate timecourses, whilst in other cases a single region may contain multiple independent signals. In future, the use of brain parcellations based directly on the MEG data may therefore prove instructive. However this is non-trivial and should be a subject of future investigation. Secondly, for source localisation, we used a beamformer technique. Beamforming has been shown previously to be particularly useful in the characterisation of neural oscillations, and has been used successfully in the measurement of connectivity (Brookes et al., 2011). The reasons for the success of this algorithm in such studies has been addressed at length in previous papers, and will not be repeated here. However, we do point out that other inverse solutions could be substituted for beamforming in the present processing pipeline, and would likely generate similar results. Thirdly, regarding the choice of functional connectivity metric: here we choose to use envelope correlation based on the previous success of this measurement in facilitating long range connectivity estimation. However, it is important to point out that the multi-layer network framework is not limited to envelope metrics, but could be extended to other electrophysiological measurements of functional connectivity. Recent years have seen the emergence of a number of metrics for functional coupling, including within frequency band and between frequency band interactions. It is easy to conceive how such metrics could be employed to form a set of super adjacency matrices similar to those employed here. For example the diagonal tiles (within frequency connectivity) could easily be generated using either the imaginary part of coherence (Nolte et al., 2004) or the phase lag index (Stam et al., 2007). When considering between frequency band interactions obviously the notion of phase coupling becomes problematic. However, one could consider measuring a fixed phase relationship between two bands where, for example, the duration taken for n cycles of frequency band one always coincides with the duration taken for m cycles of frequency band two. In addition, cross frequency interactions can also be quantified via coupling between the phase of low frequency oscillations and the amplitude of high frequency oscillations (Canolty et al., 2010, Florin and Baillet, 2015). Finally, following derivation of super-adjacency matrices, there are many ways in which to analyse those matrices statistically. Here, a simple approach was employed in which significant differences between task and rest (or patients and controls) was sought on an element by element basis. We used this approach since it allowed direct inference on both task driven networks and patient-control differences. However, more complex analyses may be highly informative: In particular, graph theoretical metrics such as algebraic connectivity have become a popular way to analyse single layer networks in neuroimaging and are equally applicable to multi-layer models. Such measures would offer summary statistics regarding changes in the efficiency of the network as a whole (e.g. algebraic connectivity reflects, loosely, a measure of synchronisability of the network). Such measures may be of significant utility in characterising task, compared to rest, or patients versus controls. Overall, it is possible to conceive multiple ways of forming and analysing a multi-layer network equivalent to that used here. This same framework will offer unique insight into how the brain employs multiple temporal scales in order to simultaneously form, and dissolve, networks of communication in the task positive and resting states.

### Insights into schizophrenia

Following testing of the multi-layer framework, we sought to further demonstrate its utility by gaining insights into the neuropathology underlying schizophrenia. Abnormalities in motor function have been noted since the earliest descriptions of schizophrenia and are a well-accepted feature of the disorder (Kraepelin, 1919). Similarly, patients with schizophrenia exhibit deficits in low-level visual function (Butler et al., 2001, Cadenhead et al., 2013, Keri et al., 2002, Slaghuis, 1998). For this reason, the visuomotor task represents a useful means by which to probe abnormalities in this debilitating disorder. Using multi-layer connectivity assessment, we observed significantly reduced alpha band functional connectivity in a network of brain regions spanning the visual cortex (Fig. 6A and 6B). Furthermore, the clinical relevance of this difference was confirmed since the magnitude of measured alpha connectivity in visual cortex inversely correlated with behavioural measures representative of the persistent features of the disease (Fig. 6C). This result adds weight to an argument that impaired connectivity is a feature of Schizophrenia. Our result is further summarised in Fig. 7 which shows *activity* within and *connectivity* between the left and right primary visual regions. First note that there is no significant difference in the magnitude of stimulus driven alpha amplitude change, between patients and controls (Fig. 7B). In agreement with this, the alpha envelope timecourses (Fig. 7A) in patients and controls are remarkably similar: both show an overall loss in amplitude during stimulation, and both show a transient dip shortly after stimulus onset and offset meaning their overall structure (and signal to noise ratio) is the same. We did observe a moderate difference in amplitude between controls and patients in a small time window at around 3 s post stimulus; however this was not found to be significant (p < 0.05) following FDR correction across independent time samples. When measuring connectivity (envelope correlation) between left and right visual cortices we observed a significant reduction in the patient group (Fig. 7C right hand plot). This difference is due neither to altered leakage in patients, nor to altered SNR (see Appendix A). Recall that connectivity is measured as amplitude envelope correlation within each trial individually, prior to trial averaging. Our result thus shows that in unaveraged data, there is greater coordination (correlation) between the visual areas in controls compared to patients. Put another way, there are signal components – asynchronous across regions – which occur in patients and not in controls. Importantly, these additional signals are not task related and therefore average out across trials, since they have no observable impact on trial averaged alpha envelope timecourses. Further, there is no significant difference between groups when “connectivity” is measured using trial averaged data (Fig. 7C; left hand plot). This key point shows the importance of measuring connectivity between areas using unaveraged data.

It is important to remember that this is an exploratory analysis in a small group (23 controls and 23 patients). For this reason, results should not be over interpreted and they require replication in a second patient cohort. However, given the relatively well characterised role of alpha oscillations it is tempting to speculate on what these measurements might imply. Our multi-layer network model captured connectivity across the entire 8–100 Hz frequency range. This analysis encompassed many pan-spectral networks including the beta band sensorimotor network and the gamma band visual network (indeed, this structure is clear in the super-adjacency matrices shown in Fig. 5). It is therefore of significant note that only the occipital alpha network demonstrated a robust relationship to schizophrenia. Visual alpha oscillations have been observed since the first EEG recordings. For many years, these effects were treated as epiphenomena, with little or no relevance to neural processing. However, in recent years important insight has been gained into the functional role of these oscillatory effects. Specifically, a link has been made between alpha activity and attention, with high alpha amplitude being thought of as a marker of inattention (Handel et al., 2011, Thut et al., 2006, Zumer et al., 2014). This is shown clearly in studies in which individual subjects are asked to switch their attention from one visual region to another. If, for example, attention is switched from the left visual field to the right, one sees an increase in alpha oscillations in the right hemisphere and a decrease in the left. The reverse is true when switching attention from the right visual field to the left. Furthermore it has been proposed that these alpha oscillations act to gate information flow to higher order cortical regions (Zumer et al., 2014). Given this hypothesis, it follows that a lack of coordination between alpha envelopes across brain regions may be reflective of an inability to direct visual attention appropriately, and more specifically an inability to accurately gate incoming visual information to higher order brain regions. This, in turn, may have an influence on a number of the ongoing persistent symptoms of schizophrenia including an apparent disorganization or impoverishment of mental activity. We therefore speculate that this may be why reduction in alpha connectivity correlates well with behavioural measures of persistent illness severity. For this reason, whilst this remains an exploratory analysis, future studies of schizophrenia patients using MEG should use this same technique to further probe alpha band attentional effects and their relationship to the core symptoms of schizophrenia.

## Conclusion

We have combined oscillatory envelope based functional connectivity metrics with a multi-layer network model in order to derive a complete picture of connectivity within and between oscillatory frequencies. We demonstrate our methodology in a visuomotor task, highlighting the simultaneous and transient formation of motor networks in the beta band and visual networks in the high gamma band, as well as cross-spectral interactions. More importantly, we employ our framework to demonstrate significant differences in occipital alpha band networks in patients with schizophrenia relative to controls. We further show that these same measures correlate significantly with symptom severity scores, highlighting their clinical relevance. Our findings demonstrate the unique potential of appropriately modelled MEG measurements to characterise neural network formation and dissolution. Further, we add weight to the argument that dysconnectivity is a core feature of the neuropathology underlying schizophrenia.

## Figures and Tables

**Fig. A1:**
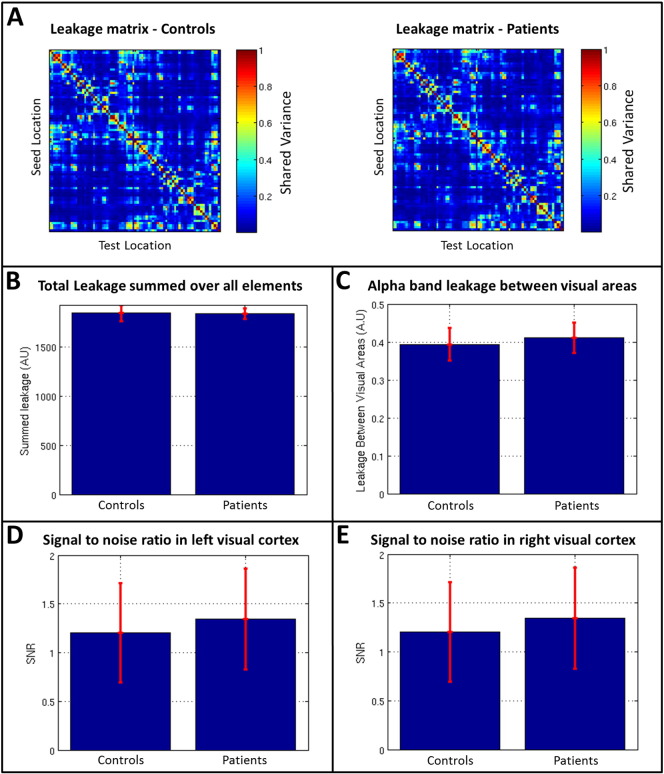
Testing for leakage and signal to noise ratio between control and patient groups. A) Adjacency matrices representing source leakage between AAL regions. Note that whilst leakage is apparent, there is little difference between groups. B) Summed leakage between all brain regions for controls and patients. C) Regional specific leakage between left and right visual cortex. D) Measured SNR in left visual area. E) Measured SNR in right visual area. Note neither leakage nor SNR differs between groups.

## Appendix A Testing signal leakage and signal to noise ratio

As noted in section 2.2, signal leakage between source space estimates is a significant problem in MEG functional connectivity estimation (Brookes et al., 2012, Colclough et al., 2015 ; Hipp et al., 2012, O'Neill et al., 2015a). Leakage results from the ill-posed MEG inverse problem, which causes spatial blurring in beamformer reconstruction and means that a point source will spread across a finite volume. In addition, mislocalisation of sources, for example due to inaccuracies in modelling the forward vector, or deviation from the assumptions driving the inverse model also adds to the effect of leakage. Overall the effect is that if two temporally independent and spatially separated sources are reconstructed via beamforming, the resultant estimated timecourses may no longer be orthogonal. This causes artifactually inflated functional connectivity estimates.

In the present paper, we employed orthogonalisation (via regression) in order to limit the effect of leakage. However this technique, whilst highly effective, is not perfect and a degree of leakage can remain if, for example, underlying beamformer projected data are non-Gaussian (O'Neill et al., 2015a). For this reason, it is conceivable that the between group differences in functional connectivity (effect of diagnosis) observed in our schizophrenia study could result directly from between group differences in leakage. Leakage itself is dependent on beamformer accuracy, and this relates directly to the accuracy of both the data covariance matrix, and the forward field model. Covariance and forward model accuracy, in turn, both rely heavily on subjects remaining still throughout the MEG data acquisition, as well as on the signal to noise ratio of the data and the total amount of data available. Patients are more likely to move during acquisition. Furthermore, patients could potentially have more eye movements, and more muscle artifact. It is therefore conceivable that patients could exhibit a different leakage profile to controls. In addition, it has been shown previously (Schoffelen and Gross, 2009) that change in connectivity can be driven by a change in SNR. Again, SNR in MEG could potentially differ between patients and controls. For these reasons it is critical in studies of this type to analyse both the leakage profile (before orthogonalisation) and SNR, measured across both groups, to check that any observable connectivity differences do not result from poor beamformer reconstructions.

Fig. A1 shows results of post-hoc tests for between group differences in leakage and SNR. Fig. A1A shows adjacency matrices representing the source leakage between all AAL region pairs. Source leakage is quantified as the fraction of variance explained in the test timecourse by the seed timecourse, prior to leakage reduction or envelope calculation. (In other words, leakage is quantified as the squared zero-phase-lag correlation between projected timecourses.) The left hand matrix shows the case for controls and the right hand matrix shows patients. Note that whilst leakage is apparent in both groups, there is little difference between them. Fig. A1B shows summed leakage (sum of all elements of both matrices in Fig. A1A) for controls and patients and Fig. A1C shows regional specific leakage between left and right visual cortex; again there is no apparent difference between groups. Finally, Fig. A1D and E show measured SNR in the left and right visual regions respectively. SNR is quantified as the mean difference in alpha band amplitude between a stimulus [0 s < t < 2 s] and control [6.5 s < t < 8.5 s] window, divided by the standard deviation of the trial averaged envelope in the control window. Again that there is no measurable difference between patients and controls. These analyses are extremely important since they show clearly that the between group differences presented in the main manuscript cannot be driven by either leakage or SNR. Such post-hoc checks should be a significant part of all future studies seeking a between group difference in MEG derived functional connectivity.
